# Context-Dependent Requirements for FimH and Other Canonical Virulence Factors in Gut Colonization by Extraintestinal Pathogenic Escherichia coli

**DOI:** 10.1128/IAI.00746-17

**Published:** 2018-02-20

**Authors:** Colin W. Russell, Brittany A. Fleming, Courtney A. Jost, Alexander Tran, Alan T. Stenquist, Morgan A. Wambaugh, Mary P. Bronner, Matthew A. Mulvey

**Affiliations:** a University of Utah School of Medicine, Department of Pathology, Division of Microbiology and Immunology, Salt Lake City, Utah, USA; b Department of Pathology, ARUP Laboratories, University of Utah, Salt Lake City, Utah, USA; c Huntsman Cancer Institute, University of Utah, Salt Lake City, Utah, USA; The University of Texas at Austin

**Keywords:** *Escherichia coli*, ExPEC, FimH, colonization, evolution, intestinal, microbiota, mouse model, uropathogenic, virulence factors

## Abstract

Extraintestinal pathogenic Escherichia coli (ExPEC) acts as a commensal within the mammalian gut but can induce pathology upon dissemination to other host environments such as the urinary tract and bloodstream. ExPEC genomes are likely shaped by evolutionary forces encountered within the gut, where the bacteria spend much of their time, provoking the question of how their extraintestinal virulence traits arose. The principle of coincidental evolution, in which a gene that evolved in one niche happens to be advantageous in another, has been used to argue that ExPEC virulence factors originated in response to selective pressures within the gut ecosystem. As a test of this hypothesis, the fitness of ExPEC mutants lacking canonical virulence factors was assessed within the intact murine gut in the absence of antibiotic treatment. We found that most of the tested factors, including cytotoxic necrotizing factor type 1 (CNF1), Usp, colibactin, flagella, and plasmid pUTI89, were dispensable for gut colonization. The deletion of genes encoding the adhesin PapG or the toxin HlyA had transient effects but did not interfere with longer-term persistence. In contrast, a mutant missing the type 1 pilus-associated adhesin FimH displayed somewhat reduced persistence within the gut. However, this phenotype varied dependent on the presence of specific competing strains and was partially attributable to aberrant flagellin expression in the absence of *fimH*. These data indicate that FimH and other key ExPEC-associated factors are not strictly required for gut colonization, suggesting that the development of extraintestinal virulence traits is not driven solely by selective pressures within the gut.

## INTRODUCTION

The bacterium Escherichia coli was first described by Theodor Escherich in 1884 and has since become a critical model organism that has been used to understand the fundamentals of molecular biology (1). E. coli is able to live in a variety of locations, including the soil, water, and the human gut. Although it is a prominent member of the neonatal microbiota, it is quickly overshadowed by burgeoning anaerobic bacteria as oxygen becomes scarce within the gut following birth (2, 3). As the intestinal microbiota develops, E. coli and other facultative anaerobes preferentially colonize the mucus layer that lines the large intestine, where oxygen is most abundant (around 40 mm Hg) (1, 4). In contrast, most anaerobic bacteria occupy the lumen, where oxygen is scarce (typically less than 1 mm Hg) (4). In adults, E. coli is present at about 10^7^ to 10^9^ CFU/g feces, a level that is 100- to 10,000-fold lower than that of the resident anaerobes (5). Despite being a minor component of the microbiota, the estimated number of E. coli cells that are transmitted via fecal matter from each human being to the environment in a single day is staggering: about 10^11^ CFU (6).

Understanding the role of E. coli within the microbiota is complicated by the fact that E. coli is a very diverse species with a wide spectrum of phenotypes (1). Some E. coli strains live harmlessly in the gut, while others act as pathogens, causing diarrhea and hemorrhaging (7). A few strains have been linked with the development of Crohn's disease and colorectal cancer (8–10). One strain, Nissle 1917, acts as a probiotic that assuages inflammation in addition to inhibiting colonization by pathogens such as Salmonella (11, 12). A group of strains known as extraintestinal pathogenic Escherichia coli (ExPEC) generally acts as commensals within the gut but can disseminate to other host environments and subsequently cause disease (13). ExPEC includes uropathogenic E. coli (UPEC), which causes the overwhelming majority of urinary tract infections (UTIs) (14). These infections are especially prevalent among women, about half of whom will have at least one UTI during their lifetime. ExPEC is also responsible for other, more serious conditions, including sepsis and neonatal meningitis (13, 15).

The gut is thought to be the major ExPEC reservoir that seeds extraintestinal infections. Evidence for this notion is that the same ExPEC strain can often be isolated from both the feces and urine of individual patients suffering from UTIs (16–19). Indeed, ExPEC strains are frequently difficult to clear from the gut with antibiotic treatments, even when the pathogens are effectively eliminated from the urinary tract (20). Furthermore, E. coli strains belonging to phylogenetic group B2, which includes many ExPEC isolates, are much more likely to be long-term residents within the gut than are other E. coli populations (21–23). The majority of adults carry group B2 E. coli strains within the gut, irrespective of extraintestinal infections (23). Cumulatively, these observations suggest that ExPEC primarily inhabits the gut, with sporadic departures to extraintestinal sites.

Given that ExPEC resides mostly within the gut and that the transmission of ExPEC among individuals likely occurs chiefly through fecal-oral routes (5, 24–26), it is expected that ExPEC genomes have been shaped in large part by the evolutionary pressures present within the gut. How, then, did extraintestinal virulence factors come into being? The hypothesis of coincidental evolution has often been evoked to answer this question (27). In general terms, coincidental evolution is when a factor evolves in one context but happens to be useful in another context as well (28). When the hypothesis of coincidental evolution is applied to the ExPEC life cycle, the implication is that factors that promote virulence in extraintestinal niches evolved in the gut for a function possibly unrelated to virulence.

Little concrete evidence has been put forth to support or contradict coincidental evolution in the context of ExPEC infection, other than the fact that known extraintestinal virulence factors are often encoded by gut isolates (22, 27, 29). One prediction of this hypothesis is that extraintestinal virulence factors should play a role in gut colonization. To date, this possibility has been addressed by only one study, in which an ExPEC mutant that lacks multiple pathogenicity-associated islands (PAIs) was found to be defective in its ability to persist within the murine intestinal tract (6). It is clear that more experimental work needs to be done to determine the extent to which coincidental evolution applies to single virulence factors within ExPEC. To address this issue, several canonical virulence factors were individually deleted from a reference ExPEC isolate, and the resulting mutants were tested for their ability to colonize and persist within the mouse gastrointestinal tract. Among eight virulence factors that were examined, only the type 1 pilus-associated mannose-binding adhesin FimH had a notable persistence defect within the gut. However, this defect was variable and partly contingent upon aberrant flagellin expression by the *fimH* mutant and the presence of specific competing strains. These findings are discussed in the context of both coincidental evolution and the development of anti-ExPEC therapeutics.

## RESULTS

### ExPEC stably colonizes the murine intestinal tract in the presence of the natural, intact microbiota.

**FIG 1:**
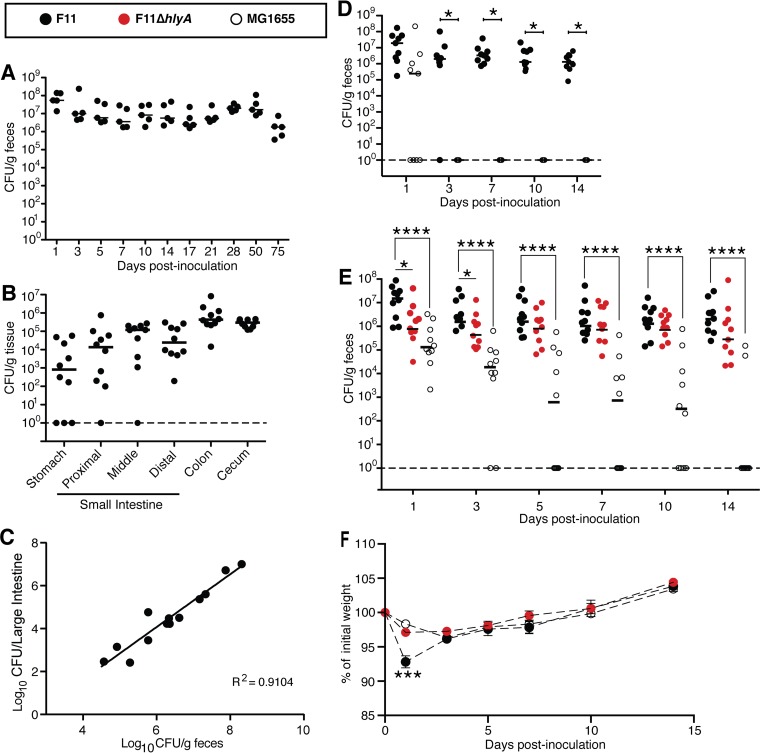
ExPEC colonizes and persists within the gut of SPF mice without causing serious long-term pathology. Adult female SPF BALB/c mice were inoculated via oral gavage with ∼10^9^ CFU of bacteria. (A) Titers of F11 recovered from the feces of mice at various time points postgavage (*n* = 5 mice). (B) F11 titers within tissues from the intestinal tract at 14 days postgavage. (C) F11 titers within the feces relative to titers within the large intestines (colon with cecum and associated fecal matter), determined at 24 h postgavage (*n* = 13). (D) Mice were gavaged with a 1:1 mixture of F11 and MG1655, and fecal titers were determined for both populations at the indicated time points. *, *P* < 0.05 by Wilcoxon signed-rank tests with corrections for multiple comparisons. (E) Fecal titers from noncompetitive assays in which mice were orally inoculated with F11, F11Δ*hlyA*, or MG1655. ****, *P* ≤ 0.0001 by Mann-Whitney U tests with corrections for multiple comparisons; *, *P* < 0.05 by Mann-Whitney U tests, only without corrections. In panels A to E, bars indicate median values. (F) Relative weights (mean values ± standard deviations) of mice following oral inoculation with F11, F11Δ*hlyA*, or MG1655. Data were normalized to the mass of each mouse prior to gavage. ***, *P* ≤ 0.005 with corrections when F11 is compared to either F11Δ*hlyA* or MG1655 by multiple *t* tests (in panels B to F, *n* ≥ 10 mice from at least two independent experiments).

To examine ExPEC colonization of the intestinal tract, we employed a model in which adult specific-pathogen-free (SPF) BALB/c mice were inoculated with ∼10^9^ CFU of the reference cystitis isolate F11 by intragastric gavage. At various time points postgavage, feces were collected, and the numbers of viable ExPEC bacteria present were enumerated. F11 and other bacterial strains used in this study were engineered to express either chloramphenicol resistance (Clm^r^) or kanamycin resistance (Kan^r^) cassettes so that they could be easily identified by plating fecal homogenates onto selective medium. Following inoculation, the fecal titers of F11 remained fairly stable for up to 75 days, with median values ranging between 10^6^ and 10^7^ CFU/g feces after the first day (Fig. 1A). These data demonstrate that ExPEC can efficiently initiate and maintain colonization of the SPF mouse gut, in line with data from recent reports from our group and others (25, 30–32). Consistent with the observation that nonpathogenic E. coli resides mostly within the large bowel (33), the cecum and colon carried the largest load of F11 at the 2-week time point, although considerable numbers of F11 bacteria were also present within the small intestine (Fig. 1B). Relatively few bacteria were recovered from the stomach, which is not thought to be a stable niche for E. coli. Due to the coprophagic nature of mice, it is likely that the animals are ingesting fecal material containing shed F11 and that the low numbers of F11 bacteria recovered from the stomach simply represent bacteria that are in transit to the small intestine. The amounts of F11 bacteria shed in the feces did not allow efficient intestinal colonization via mouse-to-mouse transmission through coprophagy (C. W. Russell, B. A. Fleming, and M. A. Mulvey, unpublished data). Importantly, in our assays, F11 fecal titers correlated well with the levels of F11 detected within the lower intestinal tract (Fig. 1C), indicating that the enumeration of fecal titers is a valid proxy for assessing gut colonization.

It is of note that the SPF mice utilized in these experiments were not treated with antibiotics, and therefore, each mouse possessed an intact microbiota. This is in contrast to other commonly used mouse models in which mice are treated with streptomycin and/or other antibiotics in order to disrupt the intestinal microbiota and open up niches that can then be occupied by incoming microbes (33). Since antibiotic treatment was not required for consistent colonization of the gut by F11 (or by other ExPEC isolates [25, 30]), we wondered whether ExPEC is simply more adept at gut colonization than nonpathogenic E. coli strains. As a test of this idea, we first competed F11 head-to-head with MG1655, an often-used nonpathogenic E. coli K-12 strain. Following oral gavage with equal numbers of F11 and MG1655 bacteria, the K-12 bacteria were cleared from all of the mice by day 3, while F11 stably persisted (Fig. 1D). When mice were inoculated with MG1655 alone by gavage, it was lost at a lower rate but was still cleared from 80% of the mice by day 14 (Fig. 1E). This is in sharp contrast to the persistent phenotype observed with F11. Similar results were obtained in competitive assays using SPF C57BL/6 mice (see Fig. S1A in the supplemental material).

To assess whether ExPEC colonization of the colon is marked by inflammation (colitis), the mice were weighed at several time points after colonization, as weight loss frequently accompanies colitis. Mice that were inoculated with F11 by gavage consistently experienced transient weight loss at the 1-day time point, in comparison to mice that were inoculated with MG1655 (Fig. 1F). Since the ExPEC-associated pore-forming toxin alpha-hemolysin (HlyA) was previously shown to induce inflammation within the gut in other mouse models (34–36), we hypothesized that HlyA contributes to the transient weight loss seen in F11-colonized animals. When mice were colonized with F11Δ*hlyA*, fecal titers of the mutant were notably lower than those of wild-type (WT) strain F11 at 1 and 3 days postinoculation (uncorrected *P* values of 0.015 and 0.023, respectively) (Fig. 1E). However, these differences were not significant when *P* values were adjusted for multiple measurements. At later time points, the Δ*hlyA* mutant persisted at levels more like those of the WT strain, although the median titers of the mutant were always lower. These data, coupled with the observation that F11Δ*hlyA* does not elicit transient weight loss by the host (Fig. 1F), suggest that HlyA both enhances initial colonization of the gut by F11 and stimulates short-term inflammation that causes transient weight loss. By the 14-day time point, the colons of mice that were inoculated with WT F11, F11Δ*hlyA*, or MG1655 by gavage appeared unperturbed, having normal crypt architecture and no evidence of infiltrating immune cells, as assessed by histological analysis. Altogether, these data indicate that F11 colonization induces transient inflammation of the intestinal tract with coordinate weight loss by the host. HlyA can facilitate these processes but is not strictly required for the longer-term persistence of F11 within the gut.

### Not all ExPEC-associated toxins promote ExPEC fitness within the gut.

**FIG 2:**
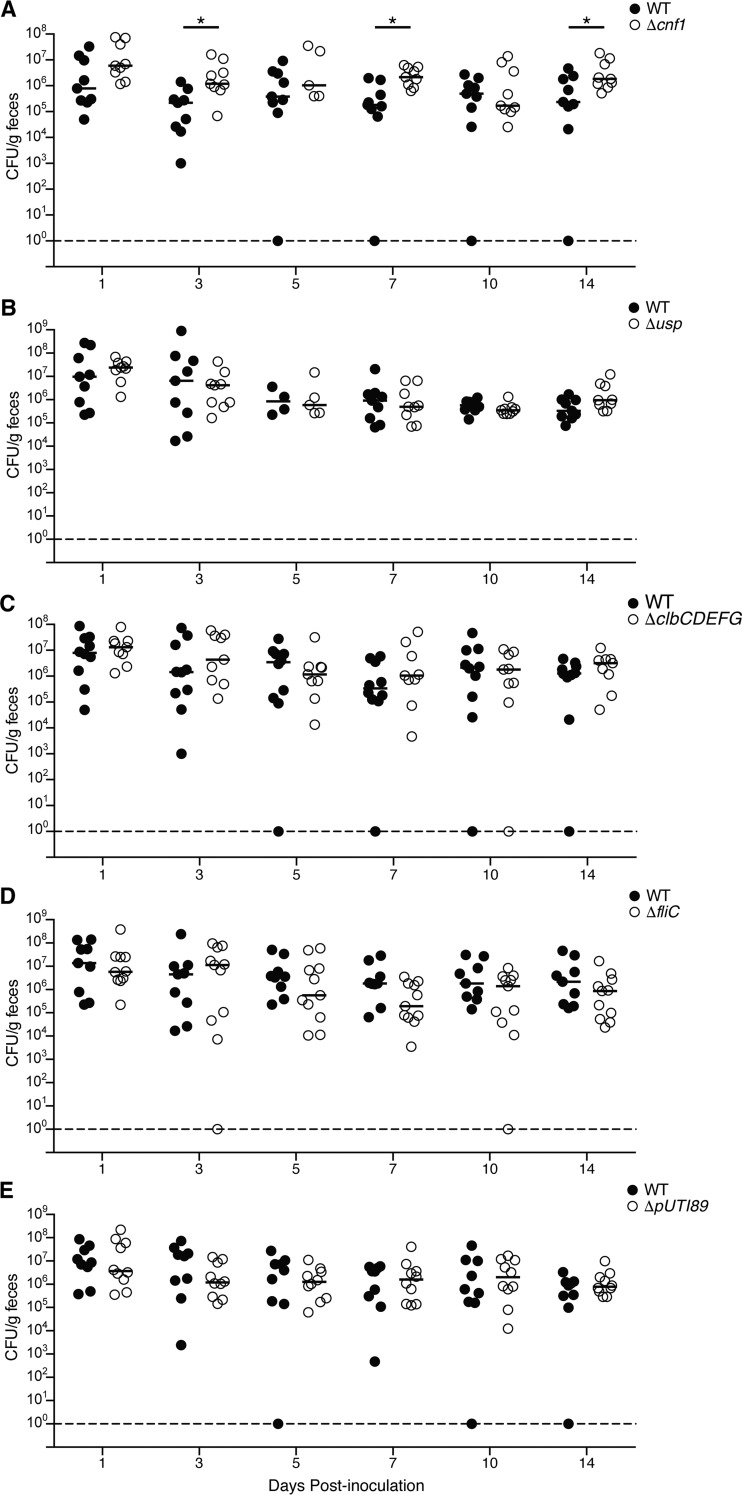
Key ExPEC-associated factors are not required for gut colonization. Mice were inoculated with ∼10^9^ CFU of WT F11 or isogenic mutant strains lacking *cnf1* (A), *usp* (B), *clbCDEFG* (C), *fliC* (D), or plasmid pUTI89 (E) by oral gavage. At the indicated time points, feces were collected, homogenized, and plated onto selective medium to determine bacterial titers. Bars represent median values (*n* = 9 to 11 mice from two independent experiments). *, *P* < 0.05 by Mann-Whitney U tests. In panel A, the *P* value at the 14-day time point is not significant when adjusted for multiple comparisons.

In light of the hypothesis of coincidental evolution and observations showing that HlyA can enhance initial colonization of the gut by F11 (Fig. 1E), we wished to examine possible roles for other canonical ExPEC-associated toxins as mediators of intestinal colonization. The first of these was cytotoxic necrotizing factor type 1 (CNF1), a secreted toxin that catalyzes the deamidation of a specific glutamine residue within Rho family GTPases (37). This causes the constitutive activation of Rho GTPases, leading to aberrant host cytoskeletal rearrangements and multinucleation (38, 39). CNF1 has been linked with ExPEC strains in epidemiological studies (39) and can enhance host inflammatory responses and ExPEC virulence in mouse models of UTI (40) and prostatitis (41). However, the effects of CNF1 on ExPEC fitness within the host remain unclear, clouded somewhat by conflicting reports (40–42). To test if CNF1 plays a role in the gut, mice were inoculated with 10^9^ CFU of either WT F11 or F11Δ*cnf1* by gavage, and intestinal colonization levels were tracked by homogenizing and plating feces at various time points. Median bacterial titers for both the WT and mutant strains ranged between 10^5^ and 10^6^ CFU per g of feces over the course of 2 weeks, with median levels of F11Δ*cnf1* being markedly higher at a few time points (Fig. 2A). These results suggest that the absence of *cnf1* can actually benefit the survival of F11 within the mouse gut.

Like HlyA and CNF1, uropathogenic specific protein (Usp) is often encoded by ExPEC isolates (43, 44). Usp is also associated with E. coli fecal isolates that are capable of long-term persistence within the human infant intestinal tract (45). Interestingly, Usp has genotoxic nuclease activity as well as homology to colicins, a group of toxins that can be used by bacteria to harm competing microbes (46–48). Given that interbacterial competition within the gut is commonplace, we tested whether Usp is important for ExPEC gut colonization. The F11Δ*usp* mutant did not exhibit any notable defects within the gut, indicating that Usp is not required in this niche (Fig. 2B).

Another toxin that has been linked to ExPEC pathogenesis is colibactin, which is produced by a number of factors encoded by the polyketide synthase (*pks*) genomic island. The *pks* island is not typically carried by intestinal pathogenic E. coli strains but is enriched in extraintestinal isolates relative to commensal fecal isolates (49). Colibactin induces DNA damage and cell cycle arrest in host cells (49, 50), and the presence of colibactin-producing bacteria has been linked with the development of colorectal cancer (51). In extraintestinal infections, colibactin-deficient bacterial strains are not as virulent as their WT counterparts. Mutation of the *pks* island reduces ExPEC translocation from the gut in neonatal rats (52) and reduces lymphopenia in septic mice (53). Whether the *pks* island also plays a role in bacterial fitness during gut colonization is not clear. Whereas one study observed decreased colonization of the small intestines of neonatal rats by colibactin mutants (52), another study found no colonization differences between a *pks* mutant and WT bacteria (54). To test whether colibactin production is important in our adult mouse model of gut colonization, we orally inoculated mice with either WT F11 or F11Δ*clbCDEFG*, which lacks a key operon within the *pks* island (55). There was no difference in the colonization abilities of the WT and mutant strains (Fig. 2C), suggesting that colibactin biosynthesis does not contribute to bacterial fitness in this model of gut colonization.

### Flagella are not required for ExPEC gut colonization.

Flagella are filamentous organelles comprised of polymers of the flagellin protein FliC (56). Although best known for their role in motility, flagella can also promote bacterial attachment and biofilm formation and can potently stimulate host inflammatory responses (57, 58). Several studies have provided evidence that flagella enhance ExPEC colonization of the mouse urinary tract (59–62). In contrast, flagella are not critical for gut colonization by nonpathogenic E. coli in streptomycin-treated mice (63). To determine if flagella are required for gut colonization by ExPEC in the face of an intact microbiota, we inoculated adult BALB/c mice with WT F11 or an isogenic mutant lacking *fliC*. In these assays, no statistical differences between the WT strain and F11Δ*fliC* were observed, although median titers for the mutant were lower at many of the time points assayed (Fig. 2D). These data suggest that the deletion of *fliC* does not have a large influence on ExPEC fitness in the gut.

### The pUTI89 plasmid is not important for gut colonization.

Many ExPEC isolates, including F11, carry plasmids that are identical or closely related to the pUTI89 plasmid that was first identified in the cystitis isolate UTI89 (64, 65). This plasmid, which is roughly 114 kb in length, harbors conjugation machinery, numerous hypothetical genes, and several putative virulence factors such as the SenB enterotoxin and the *cjrABC* operon, which may encode an iron uptake system (64). The loss of pUTI89 impairs the fitness and intracellular growth of UTI89 during the early stages of UTI in adult mice (66). Likewise, the deletion of a closely related plasmid from the neonatal meningitis E. coli isolate RS218 attenuates bacterial virulence during systemic infections in rat pups (65). In addition, a number of pUTI89-associated genes have been linked with ExPEC mucus and glucose metabolism *in vitro* (31). To determine if pUTI89 facilitates gut colonization by F11, an F11 derivative that was cured of the plasmid was tested in our mouse model. Although median titers of the cured strain were lower than those of WT F11 at early time points, no statistically significant differences between the two strains were discerned (Fig. 2E). These data suggest that pUTI89 is, for the most part, dispensable for ExPEC fitness within the gut.

### Disruption of *fimH* reduces gut colonization fitness, whereas the lack of *papG* has no effect.

**FIG 3:**
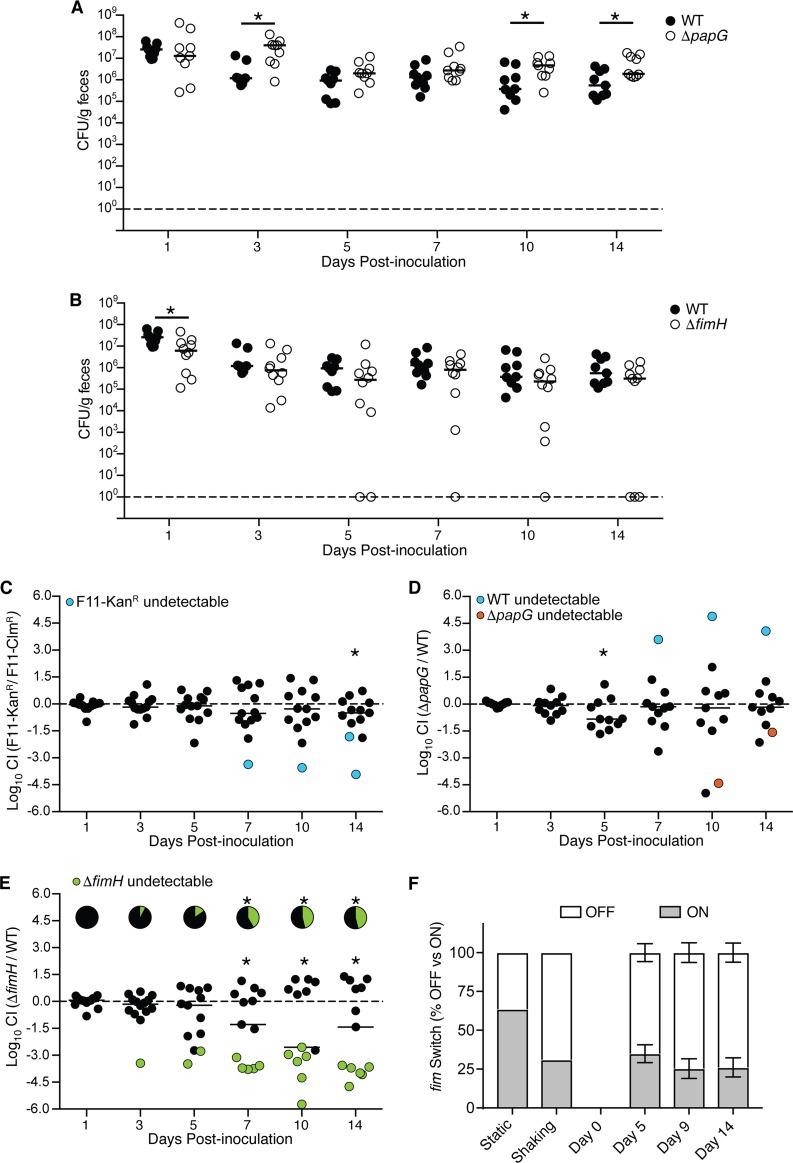
Persistence of F11Δ*fimH*, but not F11Δ*papG*, within the gut is impaired in competitive assays. (A and B) Mice were inoculated with WT F11, F11Δ*papG* (A), or F11Δ*fimH* (B) by gavage, and fecal titers were determined at the indicated time points. *, *P* < 0.05, by Mann-Whitney U tests in these noncompetitive assays. (C to E) For competitive assays, mice were inoculated with a 1:1 mixture of F11-Kan^r^ and F11-Clm^r^ (C), WT F11 and F11Δ*papG* (D), or WT F11 and F11Δ*fimH* (E) by gavage. *, *P* < 0.05 by one-sample *t* tests. Pie charts in panel E indicate the fractions of mice in which F11Δ*fimH* was not detected. *, *P* < 0.05 by Fisher's exact tests without corrections (in panels A to E, horizontal bars denote median values; *n* = 11 to 13 mice from two independent assays). For all data in panels A to E, only the *P* value for the 3-day time point in panel A is significant when corrections are made for multiple comparisons. (F) Graph showing fractions of the *fim* switch in the on and off positions from fecal samples recovered from mice following oral inoculation with F11. Bars indicate mean values ± standard errors of the means (*n* = 5 to 10 mice). Results from F11 grown in static or shaking LB broth are shown for comparison.

ExPEC strains encode many types of hair-like adhesive organelles known as pili, or fimbriae. Two of the most often-studied ExPEC-associated adhesins are P pili and type 1 pili (T1P) (67). P pili terminate with the PapG adhesin, which binds host globoseries glycosphingolipids and can facilitate bacterial infection of the kidneys. T1P are capped by the mannose-binding adhesin FimH, which promotes biofilm formation as well as bacterial attachment to and invasion of bladder epithelial cells. In our noncompetitive gut colonization assays in which the WT and mutant bacterial strains were kept separate, the deletion of either *papG* or *fimH* did not impair the ability of F11 to colonize the intestinal tract (Fig. 3A and B). Interestingly, like the Δ*cnf1* mutant, F11Δ*papG* fared somewhat better than the WT strain at days 3, 10, and 14 postinoculation. Notably, in these assays, the Δ*fimH* mutant was cleared in 3 out of 10 mice, more than what was observed with any of the other tested mutants (Fig. 3B). These results prompted us to examine the *fimH* mutant further using competitive assays in which the WT and mutant strains were inoculated as a 1:1 mixture into mice via oral gavage. In these and other competitive assays, total ExPEC levels remained fairly steady over time at around 10^6^ CFU/g feces, even when ratios of the individual competing ExPEC strains were variable.

As a control, we first competed Kan^r^- and Clm^r^-tagged F11 strains against one another to assess if the resistance cassettes alone compromised bacterial fitness within the intestinal tract. In these control assays, F11-Kan^r^ exhibited a modest defect, amounting to about a 3-fold decrease relative to F11-Clm^r^ on day 14, with a median competitive index (CI) (see Materials and Methods) of −0.49 (uncorrected *P* = 0.044) (Fig. 3C). Likewise, no striking differences between F11-Clm^r^ and F11Δ*papG* (Kan^r^) were observed in competitive assays, with the exception of a transient 6.8-fold decrease (median CI of −0.83) in the prevalence of the *papG* mutant on day 5 (uncorrected *P* = 0.038) (Fig. 3D). In contrast, when F11Δ*fimH* (Kan^r^) was competed against F11-Clm^r^, the Δ*fimH* mutant became progressively worse than the control strain starting at day 7 postinoculation (Fig. 3E). By day 10, there was about a 360-fold reduction in the relative levels of F11Δ*fimH* recovered in the feces, reflecting a median CI of −2.55 (uncorrected *P* = 0.039). In addition, the Δ*fimH* mutant began to be cleared by as early as day 3 and was not detected in the feces of nearly half of the mice by day 10 (Fig. 3E, green). In comparison, F11Δ*papG* dropped below levels of detection in only one mouse during the 14-day time course of these competitive assays (Fig. 3D, red). The phenotype observed with the Δ*fimH* mutant suggests that a disruption of this gene can markedly impair ExPEC persistence within the gut in competitive assays.

### T1P expression by F11 is modest following excretion from the gut.

T1P are phase variable, being turned on or off through the recombinase-mediated flipping of an invertible promoter element within the *fim* gene cluster (67). The orientation of this *fim* switch can be monitored and quantified by PCR as a means to assess levels of T1P expression (68, 69). Within the feces of mice that are colonized by F11, we found that the *fim* switch is in the on position in about 25 to 35% of the excreted bacteria recovered on days 5, 9, and 14 postinoculation (Fig. 3F). This is on par with results from shaking broth cultures, in which the *fim* switch is skewed toward the off position. These data indicate that T1P expression by feces-associated ExPEC is limited, which may enable the pathogen to better disseminate either within the intestinal tract or after being discharged from the host.

### Increased flagellin expression partially explains the colonization defect observed with F11Δ*fimH*.

T1P, and FimH in particular, may enhance ExPEC persistence within the gut via multiple mechanisms, such as aiding in the formation of protective biofilm-like communities or facilitating bacterial attachment to intestinal tissues (58, 70, 71). However, it is conceivable that a disruption of *fimH* could also reduce ExPEC fitness within the gut via effects on other bacterial or host processes. Specifically, previous work showed that the deletion of the entire *fim* operon in the ExPEC isolate UTI89 causes the upregulation of FliC with a coordinate increase in swimming motility (72). This could be problematic for *fim* mutants since it is known that the aberrant overexpression of FliC can impair ExPEC colonization of the gut (6), possibly as a consequence of FliC-mediated stimulation of host inflammatory responses (57). Furthermore, within the intestinal tracts of germfree or streptomycin-treated mice, mutations that reduce bacterial motility and FliC expression are selected and can promote the persistence of K-12 strain MG1655 (73–75). Together, these observations led us to question if defects in the ability of F11Δ*fimH* to survive within the gut in competitive assays are associated with altered FliC expression.

**FIG 4:**
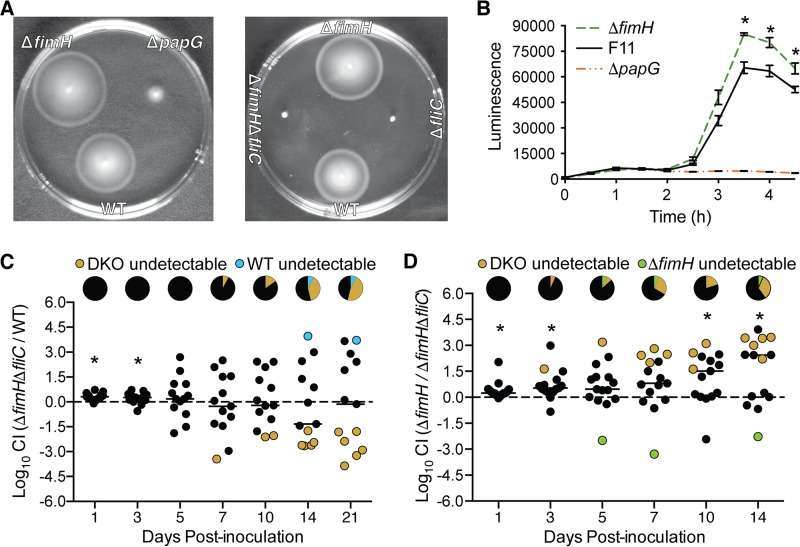
Flagellin expression impacts the efficacy of gut colonization by F11Δ*fimH*. (A) WT F11 and the indicated mutant derivatives were inoculated into motility agar to assess swimming. Images of swim plates were taken at 8 to 10 h postinoculation and are representative of results from three independent assays. (B) Plot showing results from *fliC* expression reporter assays with WT F11, F11Δ*fimH*, and F11Δ*papG* carrying p*fliC-lux*. Lines indicate mean luminescence values ± standard errors of the means from three independent assays performed in triplicate (*P* < 0.05 by multiple *t* tests). (C and D) Graphs showing results from competitive assays in which mice were inoculated with a 1:1 mixture of WT F11 and F11Δ*fimH*Δ*fliC* (DKO) bacteria (C) or F11Δ*fimH* and the DKO mutant (D) by oral gavage. Fecal titers of each strain were determined at the indicated time points by plating onto selective medium. *, *P* < 0.05 by one-sample *t* tests with corrections for multiple comparisons. Pie charts in panels C and D denote the fractions of mice in which the DKO mutant, WT F11, or F11Δ*fimH* was not detected. No significant differences were discerned by Fisher's exact tests (*n* = 13 to 15 mice from at least two independent assays). Horizontal bars indicate median values. The *P* values for data at the 3-h time point in panel B, the 1-day time point in panel C, and days 1, 3, 10, and 14 in panel D are significant when adjusted for multiple comparisons.

In assessing this possibility, we first noted that the deletion of *fimH* increases the motility of F11 in swim agar plates (Fig. 4A). Complementation of the Δ*fimH* mutant with a plasmid that expresses FimH in *trans* restored motility to WT levels (see Fig. S2 in the supplemental material). The increased motility of F11Δ*fimH* correlated with augmented FliC expression (Fig. 4B), as measured by the use of the low-copy-number reporter construct p*fliC-lux*, in which the *luxCDABE* gene cluster encoding bacterial luciferase is transcriptionally fused with the conserved *fliC* promoter (62). These results mirror those reported previously for a UTI89 mutant lacking the entire *fim* operon (72). Interestingly, in our assays, the deletion of *papG* in F11 had nearly the opposite effect of the *fimH* deletion, greatly reducing the motility of F11 and ablating FliC expression (Fig. 4A and B).

To test if FliC contributes to the defects in gut colonization observed with F11Δ*fimH*, we generated a double-knockout (DKO) mutant lacking both *fimH* and *fliC*. This mutant (F11Δ*fimH*Δ*fliC*) is nonmotile in swim plates, similar to the single Δ*fliC* mutant strain (Fig. 4A, right). In competitive gut colonization assays with WT F11, the Δ*fimH*Δ*fliC* mutant exhibited less-pronounced deficiencies than did F11Δ*fimH* (compare Fig. 4C with 3E). The greatest defect was observed at day 14 postinoculation, at which point F11Δ*fimH*Δ*fliC* was not detected in the feces of 5 of the 13 mice. Relative to the WT strain, F11Δ*fimH*Δ*fliC* titers were reduced by about 21-fold on day 14, corresponding to a median CI of −1.33 (uncorrected *P* = 0.711). This defect was substantially smaller than the maximal ∼360-fold reduction seen with the single Δ*fimH* mutant in competition with WT F11 on day 10 postinoculation (uncorrected *P* = 0.046) (Fig. 3E). In addition, clearance of F11Δ*fimH*Δ*fliC* was not observed until day 7 postinoculation (Fig. 4C), whereas the loss of F11Δ*fimH* was evident starting at day 3 (Fig. 3E). In light of the delayed defects seen with F11Δ*fimH*Δ*fliC*, we extended the assays to day 21. At this point, the median CI value returned close to 0, although F11Δ*fimH*Δ*fliC* was still undetectable in feces from nearly half of the mice, while the WT strain was present in all but one sample (Fig. 4C). Differences between F11Δ*fimH*Δ*fliC* and the WT strain at later time points were not statistically significant. The less-conspicuous defects seen with F11Δ*fimH*Δ*fliC* in these assays suggest that aberrant FliC expression contributes to the compromised persistence of F11Δ*fimH* within the gut.

### F11Δ*fimH* outcompetes F11Δ*fimH*Δ*fliC* within the gut.

To better understand the effects that the loss of *fimH* and *fliC* have on bacterial fitness during gut colonization, the Δ*fimH*Δ*fliC* and Δ*fimH* mutants were directly competed. We hypothesized that the Δ*fimH* strain, with heightened FliC expression, would be outcompeted by the Δ*fimH* Δ*fliC* DKO mutant. However, F11Δ*fimH*Δ*fliC* exhibited clear and statistically significant defects in comparison with F11Δ*fimH* (Fig. 4D). Fecal titers of the DKO mutant were below levels of detection in 1 out of 15 mice on day 3 postinoculation, and by day 14, the DKO mutant was undetectable in the feces of one-third of the animals. At this point, F11Δ*fimH* was about 270-fold more abundant than the DKO mutant, corresponding to a median CI of 2.43 (corrected *P* = 0.029). These data, as well results from noncompetitive assays (Fig. 3B), indicate that the importance of FimH to ExPEC survival within the intestinal tract is dependent upon the nature of the competing microbes that are present.

### WT F11 and F11Δ*fimH* can outcompete one another in reciprocal serial colonization assays.

**FIG 5:**
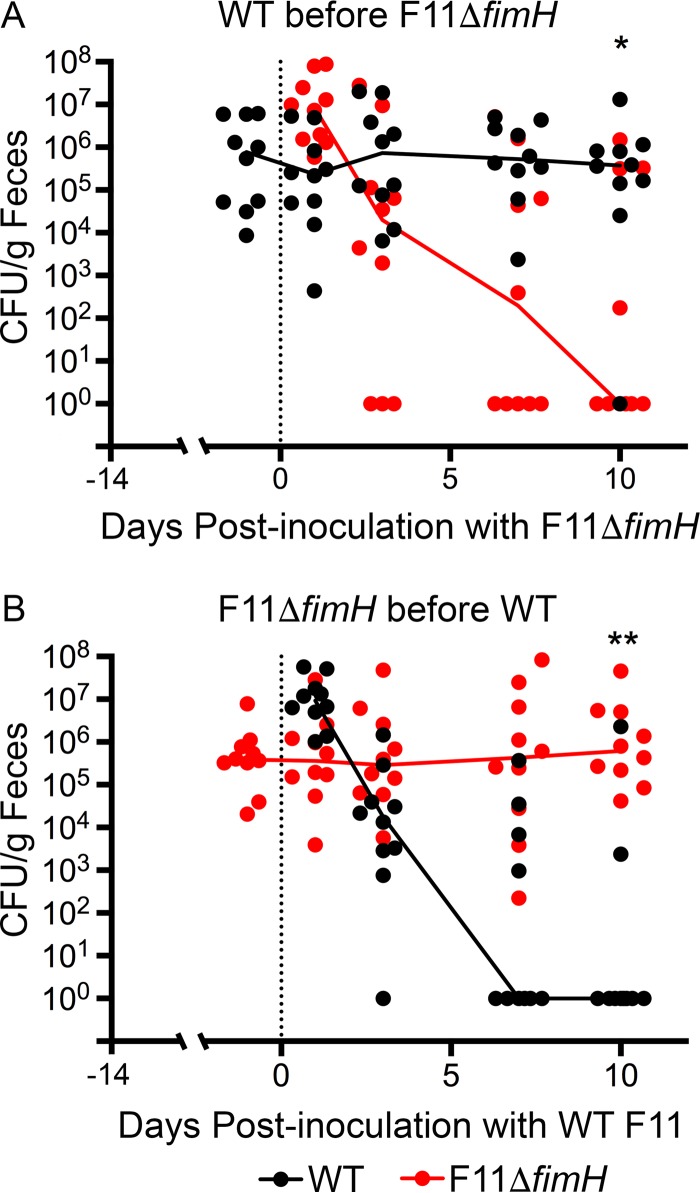
Precolonization of mice with F11Δ*fimH* effectively limits colonization by the WT strain, and vice versa. (A) BALB/c mice were inoculated via oral gavage with WT F11 (specifically F11-Clm^r^) and then with F11Δ*fimH* (Kan^r^) 14 days later. (B) Alternatively, mice were inoculated with F11Δ*fimH* followed 14 days later by the WT strain. Solid lines connect median fecal titers of each strain over time. The zero time point (dotted line) indicates when the second strain (WT F11 or F11Δ*fimH*) was introduced. At the endpoints, the *P* value was 0.0256 (*) or 0.0003 (**), as determined by Mann-Whitney U tests (*n* = 10 mice from two independent assays).

To further evaluate FimH requirements within the intestinal tract, we carried out serial colonization assays in which BALB/c mice were first inoculated with WT F11 by oral gavage before the introduction of F11Δ*fimH* 7 days later. Although fecal titers of the Δ*fimH* mutant were initially high and on par with those of the WT strain, levels of the mutant dropped precipitously and were below the limits of detection in most mice by day 3 postinoculation (Fig. 5A). In reciprocal experiments, in which F11Δ*fimH* was allowed to colonize the mice prior to the instillation of the WT strain, F11Δ*fimH* persisted, while WT F11 was cleared from most of the mice by day 10 (Fig. 5B). These data show that FimH is not strictly required for ExPEC to prevent colonization of the gut by a new competing strain, although the adhesin seems to aid in this process. In addition, these results indicate that WT F11 and F11Δ*fimH* likely vie for the same intestinal niches, with the first strain established having the upper hand irrespective of FimH expression.

## DISCUSSION

The concept of coincidental evolution, coupled with phylogenetic analyses, suggests that the extraintestinal success of ExPEC strains is a by-product of their ability to colonize the gut (27). A corollary of this hypothesis is that extraintestinal virulence and fitness factors promote ExPEC colonization of the gut. In support of this possibility, researchers previously showed that the deletion of the seven major pathogenicity islands (PAIs) of ExPEC isolate 536 not only reduced the virulence of this pathogen in a murine sepsis model but also attenuated pathogen persistence within the gut (6). Here, we set out to determine if the principle of coincidental evolution could be applied to individual virulence and fitness determinants encoded by the reference ExPEC strain F11.

In noncompetitive assays using adult SPF mice, we observed no major defects in intestinal colonization or the persistence of ExPEC mutants lacking Usp, colibactin, flagellin, or plasmid pUTI89 (Fig. 2). F11Δ*hlyA* exhibited early colonization defects, but these defects did not have a statistically significant effect on the longer-term survival of the ExPEC strain within the gut (Fig. 1E). In contrast, F11Δ*cnf1* tended to colonize the gut at somewhat higher levels than WT F11 (Fig. 2A). These results indicate that *cnf1* may negatively impact ExPEC survival in some situations, perhaps by eliciting localized inflammation within the intestinal tract. Like F11Δ*cnf1*, a mutant lacking the adhesin *papG* also colonized the murine intestinal tract at moderately higher levels than the WT strain (Fig. 3A), although in competitive assays, F11Δ*papG* had a transient colonization defect (Fig. 3D). In total, these results indicate that at least some ExPEC-linked genes can influence bacterial fitness within the gut, although the effects may be modest. Discerning more unequivocal phenotypes for individual ExPEC-associated loci within the gut can be complicated and context dependent, as exemplified by the analysis of Δ*fimH* mutants.

In noncompetitive assays, we observed no significant differences between WT F11 and F11Δ*fimH*, although the mutant was cleared from a few mice over the course of the 14-day experiments (Fig. 3B). This prompted us to test F11Δ*fimH* further using competitive assays with the WT strain. In these assays, differences between the Δ*fimH* mutant and WT strains were more distinct but not significantly so if the data are corrected for multiple comparisons (Fig. 3E). Still, the fact that F11Δ*fimH* titers within the feces from nearly half of the mice fell below detectable levels by day 10 postinoculation suggests that FimH can contribute to the persistence of ExPEC within the gut. These findings are in line with recently reported data showing that the intestinal persistence of ExPEC isolate UTI89 within streptomycin-treated mice is enhanced by FimH expression (76). That study, as well as new results with ExPEC isolate CP9 and other work with enteric E. coli pathogens (71, 77–79), indicates that FimH can promote bacterial interactions with intestinal epithelial cells. In our mouse models, we observed that F11 is localized primarily within the lumen of the colon, although substantial numbers of the pathogen are also associated with the colonic tissue (see Fig. S1B in the supplemental material). In addition to facilitating ExPEC interactions with the intestinal epithelium (76, 79), the FimH adhesin may also influence pathogen persistence within the gut via effects on other processes, including biofilm development and the modulation of innate host defenses (58, 70, 80, 81).

Defining the contributions of FimH to ExPEC fitness within the gut is further complicated by the observation that the deletion of *fimH* enhances FliC expression by F11 and increases motility (Fig. 4). Analysis of the Δ*fimH*Δ*fliC* DKO mutant in competition with WT F11 indicated that aberrant FliC expression is at least partially responsible for the colonization defects observed with the Δ*fimH* mutant (Fig. 3E and 4C). This situation mirrors results reported for ExPEC strain 536, in which the reduced intestinal persistence of the mutant lacking seven PAIs was attributed to FliC overexpression (see above) (6). The expression of flagella is generally thought to have mostly detrimental effects on E. coli fitness within the gut, possibly due to an increased burden on bacterial metabolism and the ability of FliC to stimulate host inflammatory pathways (57, 73–75). However, in competitive assays, F11Δ*fimH* outperformed F11Δ*fimH*Δ*fliC*, even though the single mutant is hypermotile (Fig. 4D). One potential explanation for this finding is that flagellum expression might at times be an advantage for E. coli within one or more intestinal niches, as previously suggested (75). This possibility is difficult to reconcile with the observation that F11Δ*fliC* has no substantial defects in our noncompetitive assays (Fig. 2D). It is plausible that cross talk among bacterial regulators of motility, T1P expression, and other adhesins also contribute to the phenotypes observed in our assays with the *fimH* mutants and other F11 derivatives (82–86), but this will require additional studies to tease apart.

The ability of distinct types of bacteria to utilize different spatial and nutritional niches within the gut allows for the coexistence of the diverse organisms that comprise the intestinal microbiota and helps provide a barrier against colonization by newly arriving microbes (87). The latter effect, known as colonization resistance, is one reason why it is generally necessary to treat mice with an antibiotic like streptomycin to open up niches for incoming bacteria that are delivered into the gastrointestinal tract by oral gavage (33). A striking feature of our experimental system is that F11 is able to colonize and persist indefinitely within the intestinal tract of conventional SPF mice without the need to first administer antibiotics (Fig. 1A). Our group and others have recently reported similar results with distinct ExPEC isolates in different mouse strain backgrounds (25, 30, 32, 88). ExPEC may be able to effectively colonize our untreated mice because they have very low numbers of endogenous E. coli bacteria and other Enterobacteriaceae based on 16S rRNA gene sequencing and selective plating assays (C. W. Russell and M. A. Mulvey, unpublished data). Nevertheless, these animals are still resistant to colonization by K-12 strain MG1655, indicating that MG1655 lacks one or more genes that ExPEC employs to persist within the gut. When in competition with F11, MG1655 is cleared from the gut at a notably higher rate than in noncompetitive assays (Fig. 1), suggesting that these two strains compete for the same intestinal niches.

A better understanding of the survival mechanisms used by ExPEC within the intestinal tract may aid in the development of more efficacious probiotics while also elucidating new therapeutic strategies to combat ExPEC before it is able to disseminate and cause disease at sites beyond the intestinal tract. The effectiveness of such approaches may be dependent on multiple variables, including timing, the makeup of the microbiota, and the presence of specific competing strains that can alter ExPEC requirements for individual fitness determinants. For example, in contrast to the situation in competitive assays, F11Δ*fimH* has no trouble colonizing the gut in noncompetitive experiments, and once established, this mutant can persist even when challenged with the WT strain (Fig. 3B and 5B). Thus, while FimH can facilitate ExPEC persistence within the gut in some settings, it is not always an absolute requirement. This conclusion may help reconcile results from older studies in which the expression of T1P was found to be unnecessary for E. coli persistence within the intestinal tracts of rodents and human infants (89–91).

Context-dependent variability in the phenotypic effects of fitness determinants like FimH may complicate treatment approaches as well as our ability to discern how life within the gut affects the evolution of ExPEC virulence traits. Data presented here indicate that the principle of coincidental evolution does not apply to all ExPEC-associated genes individually, although there are caveats to this conclusion. Our analysis of F11Δ*fimH* and F11Δ*hlyA* shows that the phenotypic effects of some genes within the gut can be modest and variable, depending on the experimental system that is used and the time points that are assayed. In future work, varying other experimental parameters (such as the inoculum dose, the genetic backgrounds of the host and ExPEC strain, and competition with other types of bacteria) could reveal additional bacterial genes and gene sets that can promote ExPEC fitness within the gut. In the present study, the lack of easily discernible phenotypes for multiple ExPEC-associated virulence genes within the gut suggests that the evolution of these loci is driven in large part by selective forces encountered outside the intestinal tract. For example, extraintestinal virulence has been correlated with the ability of ExPEC strains to resist killing by amoebae (92), while T1P expression has been linked with the transmission of ExPEC between individuals by promoting transient colonization of the oropharynx (89). In total, the results presented here show that piecing together the evolutionary history of ExPEC virulence and fitness traits is a complicated task. However, continuing efforts to resolve this problem will provide a more detailed picture of ExPEC ecology and may help identify niche-specific fitness determinants that could be attractive targets for therapeutic intervention.

## MATERIALS AND METHODS

### Bacterial strains.

Cystitis isolate F11 and K-12 strain MG1655 were genetically modified by using lambda red recombination that was facilitated by the pKM208 plasmid (93). Most of the constructs used for recombination were created by PCR using either pKD4 or pKD3 as a template to amplify a kanamycin or chloramphenicol resistance cassette, respectively, flanked by ∼40 bp of DNA with homology to the target insertion site. In some cases, longer homology regions were required, and three-part PCR was performed. This was done by PCR amplification of an antibiotic resistance cassette and regions that are upstream and downstream of the target gene. The primers used were designed to contain sections of homology to allow the three PCR products to be stitched together in a single reaction.

The pCP20 plasmid was used to remove the resistance cassette as necessary (94). To cure F11 of the pUTI89 plasmid, the *ccdAB* toxin-antitoxin system was replaced with a *tetA-sacB* construct, and the spontaneous loss of the plasmid was selected for on LB agar plates containing fusaric acid and sucrose, as explained previously (95). The strains used in this study are listed in Table 1, along with the primers used to create them. Prior to lambda red recombination, bacteria were grown with shaking in LB broth at 37°C. All growth in petri dishes was done by using LB agar supplemented with chloramphenicol (20 μg/ml), kanamycin (50 μg/ml), or ampicillin (50 μg/ml), as appropriate.

**TABLE 1:** Bacterial strains and plasmids used in this study

Strain or plasmid	Description^*a*^	Primer sequences^*b*^ used to create strain, reference, or source
Strains		
MG1655::*clm*	MG1655 with a chloramphenicol resistance cassette inserted at the *att*Tn*7* site	F: AGGATGTTTGATTAAAAACATAACAGGAAGAAAAATGCTGTGTAGGCTGGAGCTGCTTCG
		R: ATCGGTTACGGTTGAGTAATAAATGGATGCCCTGCGTAAGCATATGAATATCCTCCTTAG
F11	E. coli cystitis isolate (O6:K2:H31)	98
F11::*clm*	F11 with a chloramphenicol resistance cassette inserted at the *att*Tn*7* site	32
F11::*kan*	F11 with a kanamycin resistance cassette inserted at the *att*Tn*7* site	F: TCTGGCGTAGCCTGGGAGTTATTGCCGGATGCGATGCTGGTGTGTAGGCTGGAGCTGCTTCG
		R: TCACGTAAAAAAACGTCTAATCCGTAGACCGGATAAGAGGCATATGAATATCCTCCTTAG
F11Δ*clbCDEFG*::*clm*	F11 with the *clbCDEFG* operon replaced with chloramphenicol resistance	F1: TCGGGCGATCGATAGATTAG
		R1: CGAAGCAGCTCCAGCCTACACAGCTTGCGTATTCCATAAACTTC
		F2: TGTGTAGGCTGGAGCTGCTTCG
		R2: CATATGAATATCCTCCTTAG
		F3: CTAAGGAGGATATTCATATGCCCGTCACGCCATTTTACGT
		R3: TAATATACGCCAGTTGCCGC
F11Δ*cnf1*::*kan*	F11 with the *cnf1* operon replaced with kanamycin resistance	F: GATAAGGTGTAGTAAAATATTAATCTTCACAGAGGAGTGTGTAGGCTGGAGCTGCTTCG
		R: GGAGTAACTATAACAATGGCCAATAAATAATTTCCCGAACATATGAATATCCTCCTTAG
F11Δ*fimH*::*kan*	F11 with the *fimH* operon replaced with kanamycin resistance	F: TTATTGATAAACAAAAGTCACGCCAATAATCGATTGCATGTGTAGGCTGGAGCTGCTTCG
		R: ATGAAACGAGTTATTACCCTGTTTGCTGTACTGCTGATGGCATATGAATATCCTCCTTAG
F11Δ*fimH*Δ*fliC*::*clm*	F11 derivative in which the *fimH* gene has been deleted and the *fliC* gene has been replaced with a chloramphenicol resistance cassette	*fimH* and *fliC* single-knockout primers
F11Δ*fliC*::*kan*	F11 derivative in which the *fliC* gene was replaced with a kanamycin resistance cassette	F: ATGGCACAAGTCATTAATACCAACAGCCTCTCGCTGATCTGTGTAGGCTGGAGCTGCTTCG
		R: TTAACCCTGCAGCAGAGACAGAACCTGCTGCGGTACCTGGCATATGAATATCCTCCTTAG
F11Δ*hlyA*::*kan*	F11 derivative in which the *hlyA* gene has been replaced with a kanamycin resistance cassette	99
F11Δ*papG*::*kan*	F11 derivative in which the *papG* gene has been replaced with a kanamycin resistance cassette	F: ATGTTTTACTCGTTTAATGATAACATTTATCGTCCTCATGTGTAGGCTGGAGCTGCTTCG
		R: TTATGGCAATATCATGAGCAGCGTTGCTGAACCAGATAGTCATATGAATATCCTCCTTAG
F11Δ*usp*::*kan*	F11 derivative in which the *usp* gene has been replaced with a kanamycin resistance cassette	F: GTGGGCGATATTGTTTACCTGAGAATAATCGGTGAGAATGTGTAGGCTGGAGCTGCTTCG
		R: TTATCTCCTGTAGTGAATCTCATCGTGTAGTCTGGGGGTACATATGAATATCCTCCTTAG
F11::*kan*ΔpUTI89	Derivative of F11::*kan* which was cured of the pUTI89 plasmid by replacement of *ccdAB* with *tetA-sacB* followed by counterselection	F1: CTGTTCGTTTATTACGCCG
		R1: GATAGAGTGTCAACAAAAATTAGGAATGTCAGGCTCCGTTATACAC
		F2: TCCTAATTTTTGTTGACACTCTATC
		R2: TTAATCAAAGGGAAAACTGTCCATATGC
		F3: GCATATGGACAGTTTTCCCTTTGATTAAAGCACACCTCTTTTTGACATACT
		R3: GTTGCTATTTCTGGCTTAGTCAG
Plasmids		
pHJ20	Carries *fimH* under the control of the *tac* promoter	100
pHJ19	Same as pHJ20 except that *fimH* is positioned in the opposite orientation	101
p*fliC-lux*	Carries the *luxCDABE* gene cluster (encoding bacterial luciferase) transcriptionally fused with a conserved *fliC* promoter	62
pKM208	Encodes IPTG-inducible lambda red recombinase	93
pKD3	Template plasmid used to amplify the Clm^r^ cassette	94
pKD4	Template plasmid used to amplify the Kan^r^ cassette	94
pCP20	Flippase expression construct	94

a IPTG, isopropyl-β-d-thiogalactopyranoside.

b F, forward primer; R, reverse primer. In some cases, longer homology arms were created by three-part PCR. In these instances, three primer sets are listed.

### Mouse gut colonization.

Mice were handled in accordance with protocols approved by the Institutional Animal Care and Use Committee at the University of Utah (protocol number 15-12015), according to U.S. federal guidelines indicated by the Office of Laboratory Animal Welfare (OLAW) and described in the *Guide for the Care and Use of Laboratory Animals*, 8th ed. (96).

Prior to inoculation into mice, bacteria were grown statically from frozen stocks for 24 h at 37°C in 250-ml flasks containing 20 ml of modified M9 medium [MgSO_4_·7H_2_O (1 mM), CaCl_2_·2H_2_O (0.1 mM), d-(+)-glucose (0.1%), nicotinic acid (0.00125%), thiamine HCl (0.00165%), Casamino Acids (0.2%), Na_2_HPO_4_ (6 g/liter), KH_2_PO_4_ (3 g/liter), NH_4_Cl (1 g/liter), and NaCl (0.5 g/liter) in water]. A total of 12 ml of culture (6 ml of each culture for competitive experiments) was centrifuged at 8,000 × *g* for 10 min. Bacterial pellets were then washed once and resuspended in 0.5 ml of phosphate-buffered saline (PBS). To inoculate the mouse gastrointestinal tract, 7- to 8-week-old female SPF BALB/c or C57BL/6 mice (The Jackson Laboratory) were inoculated with 50 μl PBS containing ∼10^9^ CFU of bacteria by oral gavage. At various time points postinoculation, individual mice were briefly (3 to 15 min) placed into unused takeout boxes for weighing and feces collection. Freshly deposited feces were collected from the boxes and immediately added to 1 ml of 0.7% NaCl, weighed, and set on ice. The samples were homogenized shortly thereafter to break up the fecal pellets and then briefly centrifuged to pellet any insoluble debris. Supernatants were serially diluted and spread onto LB agar plates containing either chloramphenicol (20 μg/ml) or kanamycin (50 μg/ml) to select for growth of the relevant bacterial strains. Prior to gavage, fecal samples were analyzed to ensure that there were no endogenous bacteria present that were resistant to chloramphenicol or kanamycin. Mice were housed 3 to 5 per cage and were allowed to eat (irradiated Teklad Global Soy Protein-Free Extruded chow) and drink antibiotic-free water *ad libitum*. CIs were calculated as the ratio of mutant over WT bacteria recovered in the feces divided by the ratio of mutant over WT bacteria in the inoculum, as noted previously (31).

To determine F11 (specifically F11-Clm^r^) titers within the various regions of the gastrointestinal tract at 14 days postinoculation, mice were anesthetized via isoflurane and euthanized by cervical dislocation, and the cecum, colon, small intestine, and stomach were removed. The small intestine was divided into thirds, with the portion closest to the stomach being labeled “proximal” and the portion closest to the cecum being labeled “distal.” A part of each organ was weighed, placed into a Safe-Lock tube (Eppendorf) with three 3.2-mm stainless steel beads, and homogenized by using a Bullet blender (Next Advance). The homogenates were then serially diluted and plated onto LB agar plates containing chloramphenicol (20 μg/ml) to quantify the levels of F11-Clm^r^ bacteria present.

For histology, colon tissues were fixed in 10% neutral buffered formalin and submitted to the University of Utah Research Histology core for processing and staining with hematoxylin and eosin (H&E). Uninfected intestinal tissues and tissues recovered from mice 14 days after inoculation with either MG1655 or F11Δ*hlyA* were used for comparison. Random colon tissue sections from 5 or more mice in each experimental group were analyzed in a blind fashion by a trained pathologist (M.P.B.).

### Motility assays.

To test the swimming ability of particular strains, motility agar plates were made by pouring 25 ml of LB soft agar (0.1% agar) into a petri dish. Bacteria (1.5 μl from a culture grown overnight) were dispensed just below the surface of the plate, which was then incubated at 37°C for 5 to 10 h prior to imaging with a Stratagene Eagle Eye II system.

### Luciferase assays.

The p*fliC-lux* construct was kindly provided by the Mobley Laboratory (62). Shaking cultures of F11, F11Δ*papG*, and F11Δ*fimH* carrying p*fliC-lux* grown overnight were diluted 1:100 into 1 ml of fresh tryptone broth (Fisher Scientific) containing ampicillin (50 μg/ml). Three 100-μl aliquots of each culture were then transferred to a white 96-well polystyrene plate (Dynex Technologies) and grown statically at 37°C. Luminescent emission spectra were collected every 30 min for 4.5 h by using Gen5 software with a BioTek Synergy H1 plate reader. The instrument was set to integrate readings over 10 s using a gain of 135 and height of 1 mm. Before each reading, the plates were shaken for 1 s. Corresponding growth curves, which were obtained by taking optical density at 600 nm (OD_600_) measurements of cultures grown statically in clear 96-well polystyrene plates (CytoOne), showed that the strains used in these assays grew similarly.

### Analysis of the *fim* switch.

Quantification of the *fim* switch in the on and off positions was carried out essentially as previously described (68, 69). DNA was purified from feces by using the ZR Fecal DNA MiniPrep kit, whereas DNA was purified from broth cultures by using the Promega Wizard Genomic DNA purification kit. To skew the *fim* switch toward the on position, F11 was grown statically at 37°C in 20 ml LB broth in 250-ml Erlenmeyer flasks for 24 h, subcultured at a 1:1,000 dilution into fresh LB broth, and then incubated for another 24 h. To drive the *fim* switch toward the off position, bacteria were grown with shaking in LB broth to the exponential phase. Primers F11_fimS_F (TACCGCCAGTAATGCTGCTC) and F11_fimS_R (GTCCCACCATTAACCGTCGT) were used to amplify the *fim* switch region by PCR under the following thermocycler conditions: 95°C for 5 min and 10 cycles of 95°C for 30 s, 60°C for 20 s, and 72°C for 40 s, followed by 30 cycles of 95°C for 30 s, 56°C for 20 s, and 72°C for 40 s and ending with a 5-min incubation at 72°C. The reaction products were column purified, digested with HinfI for 1 h at 37°C, resolved in 1% agarose gels, and imaged. Bands representing the *fim* switch in the on and off positions were quantified by using ImageJ.

### Statistical analysis.

All statistical tests were carried out by using GraphPad Prism or Stata/IC-14 software, and corrections for multiple comparisons were made by using the Hochberg procedure (97). *P* values of ≤0.05 were considered significant.

## Supplementary Material

Supplemental material
