# Supplementary material for: Context-Dependent Requirements for FimH and Other Canonical Virulence Factors in Gut Colonization by Extraintestinal Pathogenic Escherichia coli
Source: Infect Immun. 2018 Feb 20;86(3):e00746-17. doi: 10.1128/IAI.00746-17 (PMC5820936; doi:10.1128/IAI.00746-17)
Supplement: Supplemental material [file supp_86_3_e00746-17__index.html]

Supplemental material 

# Context-Dependent Requirements for FimH and Other Canonical Virulence Factors in Gut Colonization by Extraintestinal Pathogenic Escherichia coli

## Supplemental material

- Supplemental file 1 -

  Fig. S1. F11, but not MG1655, effectively colonizes the intestinal tract of SPF C57BL/6 mice. Fig. S2. Complementation of F11Δ*fimH* in swim assays.

  PDF, 336K
