# Supplementary material for: Context-Dependent Requirements for FimH and Other Canonical Virulence Factors in Gut Colonization by Extraintestinal Pathogenic Escherichia coli
Source: Infect Immun. 2018 Feb 20;86(3):e00746-17. doi: 10.1128/IAI.00746-17 (PMC5820936; doi:10.1128/IAI.00746-17)
Supplement: Supplemental material [file IAI.00746-17_zii999092328s1.pdf]

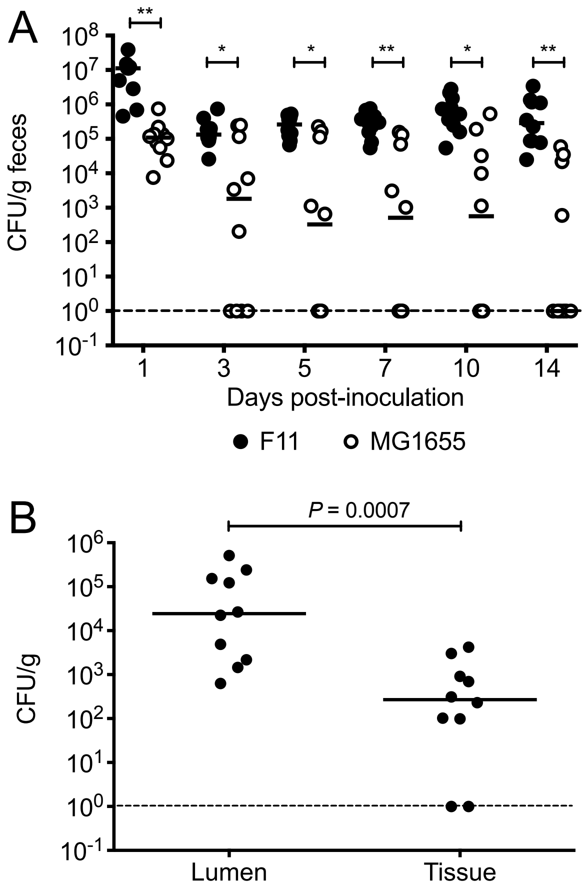

Figure S1. F11, but not MG1655, effectively colonizes the intestinal tract of SPF C57Bl/6 mice. (A) Results from competitive assays in which adult female SPF C57Bl/6 mice were inoculated via oral gavage with a 1:1 mix of F11 and MG1655 (~10^9 CFU total). (A) Graph shows titers of F11 and MG1655 recovered from the feces at the indicated time points post-gavage. n = 10 mice from two independent competitive assays. *, $P < 0.05$ and **, $P < 0.01$, as determined by Wilcoxon signed-rank tests with corrections for multiple comparisons. (B) F11 titers recovered in association with the colonic tissue or within the lumen of the colon at 14 d post-gavage. *P* value determined by Mann-Whitney test; n = 10 mice.

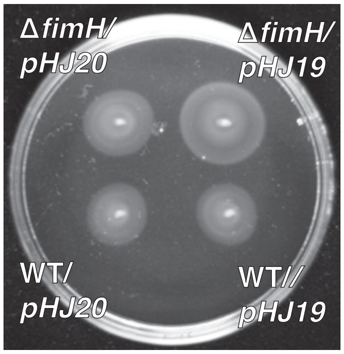

Figure S2. Complementation of F11Δ*fimH* in swim assays. WT F11 and F11Δ*fimH* carrying the indicated plasmids were inoculated into motility agar plates to evaluate swimming. The plates were imaged following 5-6 h incubations at 37°C. The plasmid pHJ20 encodes *fimH* under control of a leaky *tac* promoter. The control plasmid pHJ19 has *fimH* positioned in the opposite orientation.
